# How Health Care Organizations Approach Social Media Measurement: Qualitative Study

**DOI:** 10.2196/18518

**Published:** 2020-08-14

**Authors:** Chukwuma Ukoha

**Affiliations:** 1 Centre for Informatics and Applied Optimisation Federation University Australia Ballarat Australia

**Keywords:** health care organization, social media, measurement, benchmarking, metrics, analytics tools

## Abstract

**Background:**

Many health care organizations use social media to support a variety of activities. To ensure continuous improvement in social media performance, health care organizations must measure their social media.

**Objective:**

The purpose of this study is to explore how health care organizations approach social media measurement and to elucidate the tools they employ.

**Methods:**

In this exploratory qualitative research, Australian health care organizations that use social media, varying in size and locality, were invited to participate in the study. Data were collected through semistructured interviews, and the transcripts were analyzed using thematic analysis.

**Results:**

The study identified health care organizations’ approaches to social media measurement. While some measured their social media frequently, others used infrequent measurements, and a few did not measure theirs at all. Those that measured their social media used one or a combination of the following yardsticks: personal benchmarking, peer benchmarking, and metric benchmarking. The metrics tracked included one or more of the following: reach, engagement, and conversion rates. The tools employed to measure social media were either inbuilt or add-on analytics tools. Although many participants showed great interest in measuring their social media, they still had some unanswered questions.

**Conclusions:**

The lack of a consensus approach to measurement suggests that, unlike other industries, social media measurement in health care settings is at a nascent stage. There is a need to improve knowledge, sophistication, and integration of social media strategy through the application of theoretical and analytical knowledge to help resolve the current challenge of effective social media measurement. This study calls for social media training in health care organizations. Such training must focus on how to use relevant tools and how to measure their use effectively.

## Introduction

### Background

There is a growing use of social media in health care settings, and an increasing number of health care organizations use social media [1-4]. Social media refers to internet-based communication and interactive tools that enable the capture, storage, and presentation of written, audio, and video communication [5]. Social media is the general term for internet-based applications underpinned by the ideological and technological foundations of Web 2.0, which enables and encourages user-generated content [6] such as texts, images, and videos [7]. Social media allows users to create a profile within a bounded system, identify other users with whom they have a connection, and view and access their list of connections and those made by others within the system [8]. The International Medical Informatics Association identified 13 types of social media platforms: social networks, professional networks, thematic networks, microblogs, blogs, Wikis, forums or Listserv, social photo and video-sharing tools, collaborative filtering tools, multiuser virtual environments, social apps and games, integration of social media with health information technologies, and others (eg, FriendFeed) [9]. Social media used in health care settings can be grouped broadly into two categories—general-purpose web-based social networks and online health communities that often serve as discussion forums [10].

The ubiquitous nature of social media makes it a convenient tool for health care organizations to connect with patients and colleagues, irrespective of their geographical locations [1]. It has been predicted that social media will become the second most important form of engagement with employees and customers, second only to face-to-face interactions [11]. Social media facilitates the expansion of professional networks and participation in various professional activities, thereby giving health care practitioners a platform to express their views about the health system, which in turn helps generate and inform health policy and public debate [1]. As many as 72% of internet users have sought health information online, and many individuals regard health care organizations as their primary source of health information, in preference to advice from other sources [12].

### Objectives

Many studies [13-16] have highlighted the usefulness of social media in health care settings, and an increasing number of health care organizations are adopting the application [2-4,14]. Health care organizations use social media to support a variety of activities, including professional networking, harnessing patient feedback, public health promotion, professional education, patient education, organizational promotion, crowdsourcing, research, and patient collaboration [17].

Some health care organizations already use social media extensively, while others are relative neophytes, aiming to become “mature” social media users. Social media maturity entails a health care organization not only adopting and using the application but also possessing high levels of relevant knowledge and sophistication, along with an integrated social media strategy [18]. One of the indicators of a health care organization’s level of social media maturity is the ability to measure it [18].

Social media must be measured to ensure continuous improvement [17,19,20]. Thus, the ability to measure is critical to the success of health care social media initiatives [17,20,21]. As more forms of social media emerge, health care organizations must understand what tools to use, how to use them appropriately, and how to measure their effectiveness. The challenge for health care organizations is not just trying to find the best way to incorporate social media strategically, but also to find the best way to measure it. Against this background, this study explores how health care organizations approach social media measurement and the tools they employ.

## Methods

### Overview

This study is exploratory; thus, it follows a broadly interpretive [22] and inductive approach [23]. To solicit feedback relevant to the study’s objectives, health care organizations in Australia that use social media were invited to participate in the study.

### Ethical Considerations

In line with Federation University Australia’s ethics procedure, an ethics review form was submitted, and approval was granted on August 23, 2017. Participants were interviewed between 2017 and 2019. All participants in the study were >18 years of age. Before the interviews, all potential interviewees were allowed to read about and consent to participate in the research. Thus, participation in the study was voluntary, and no financial rewards or incentives were offered.

### Recruitment

Participant selection involved both purposive and snowball sampling. Purposive sampling involved the identification of major stakeholders [24] and ensured that initial participants were drawn from health care organizations that use social media. Initially, five hospitals that use social media were recruited to participate in the study through a combination of phone calls and emails. Apart from the initial participants, all but one of the participating organizations were recruited through snowball sampling—that is, participants suggested or helped to recruit other participants for the study. Finally, the participants were drawn from distinct types of health care organizations, varying in size and locality. Of the participating organizations, four were large hospitals that provide comprehensive health services, three were smaller hospitals that offer a wide range of medical and primary health services, and another was a medical research center. Other participants included a family practice and a clinic that promotes public health. Of the participating organizations, four were located in major cities, while the rest were located in regional areas.

Given that this study focused on health care organizations, all individuals that contribute to their organization’s social media were eligible to participate in the study. The final composition of participants was six medical doctors and five communications personnel (social media or communications managers).

### Data Collection

Qualitative data were collected through semistructured interviews, as recommended by Walsham [25]. When developing the interview questions, the researcher initially outlined the broad areas of knowledge that were considered relevant to answering the larger research questions of the study. Questions were developed within each of these areas, adjusting the language of the interview to fit participants’ backgrounds so that clinicians and communications personnel could relate to questions. The goal was to tap into their experiences and expertise. The interview guide can be found in Multimedia Appendix 1.

Participants were interviewed at their preferred time and location. In line with the process of conducting semistructured interviews, an interview guide was used flexibly [26], ensuring that conversations were free-flowing, yet focused. The flexible use of the researcher-developed interview questions enabled the interviewees to be probed further based on their responses [27]. Notes and probe questions in each interview were recorded and factored into the subsequent interviews.

Each interview was recorded with an audio recorder and then transcribed verbatim for analysis. The average duration of the interviews was approximately 50 minutes. Additional relevant information was obtained from publicly available literature about some participants in the study. After each interview transcription, the researcher carefully reviewed the transcripts and recordings to ensure that no relevant information had been missed.

The expectation was to conduct between 12 [28] and 15 interviews [29] to reach saturation of knowledge; however, after the seventh interview, the analysis of subsequent interview transcripts hardly yielded new themes. This redundancy signaled to the researcher that the data collection process might not produce additional information. In total, 11 in-depth interviews took place.

### Data Analysis

The interview data were anonymized by removing content that could identify interviewees. Utmost care was taken to preserve the richness of the interview material wherever possible, while also protecting the privacy of participants [30].

The transcript was then uploaded to NVivo (QSR International) [31] in readiness for the analysis process. Relevant qualitative data were thematically analyzed until themes emerged that helped elucidate the phenomenon under investigation.

The analysis involved the inductive development of categories. The researcher first assigned summative or evocative attributes to different portions of the transcribed interviews, identifying similarities, patterns, and relationships. There were several initial codes, with some of them overlapping to some extent. A preliminary category system was applied to the interview data. Subsequently, codes with similar meanings were clustered, and a corresponding theme was formed. The researcher modified categories when the data showed additional and new information that required a new category. The researcher differentiated the resulting defined themes into main and subcategories and assigned relevant original statements in the transcripts to these categories.

Although the coding and analysis of interview transcripts were performed solely by the researcher, the findings were reviewed with peers, including experienced researchers. Furthermore, to ensure the validity of the results, the results were considered vis-à-vis explanations from relevant literature, in line with triangulation techniques [32].

**Figure 1:**
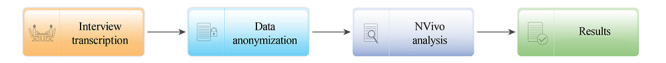
The sequence of data analysis activities.

Figure 1 presents the sequence of activities during data analysis.

## Results

### Overview

Thematic analysis of interview transcripts yielded five main themes concerning health care organizations’ approach to social media measurement: frequency of social media measurement, benchmarks used for social media measurement, metrics tracked in social media measurement, tools used in social media measurement, and challenging aspects of social media measurement.

The themes that emerged were spread over both medical doctors (MD) and communications personnel (CP).Hence, the contributions of MD and CP participants were blended and presented based on themes that emerged collectively rather than by group.

### Frequency of Social Media Measurement

Three categories emerged based on how often participants measured their social media: frequently, infrequently, and never.

#### Frequently

Some participants reported that they measured their social media frequently. This study conceptualized frequent measurement as measuring one’s social media at least once per week. In response to a question about how often they measure their social media, a participant had the following to say:

Every day. Every post that we do, we look to see how it went. So, we don’t just look at them once a month, but we look at them every day.

CP1

According to another participant:

I monitor it on a day-to-day basis and then on a weekly basis, looking back, just to see that I am on track for where I need to be for the month. Because if you don’t look at where you are until the end of the month, there’s nothing you can do to fix it if you’re not anywhere near your goal. So, it’s continuous monitoring I would say.

CP3

#### Infrequently

It was observed that some health care organizations measured their social media infrequently. This study conceptualized infrequent measurement as measuring one’s social media twice or fewer times per month. Regarding how frequently they measured their social media, a participant stated:

Not often. I give my results on a monthly report.

CP5

One reason for infrequent measurement could be that managers do not require employees who oversee the health care organization’s social media to provide social media reports frequently. In the words of a participant:

I honestly haven’t had to do that in a long while because they trust me.

CP2

Other possible reasons for not regularly measuring their social media are that some organizations do not sufficiently understand the information or are unable to afford the time, money, and effort needed.

I don’t check it all that often because, number one, you need to understand the information, number two, you need to either have the time, the money, or [afford] the effort to change that.

MD4

#### Never

Interestingly, it was noted that some health care organizations did not ever measure their social media performance. As two participants explained:

I glance at the stats. I’ve never, in all my time, ever tried to calculate, like, the stats.

MD5

I don’t measure anything. I am not sure these sorts of things can be measured.

MD6

The lack of measurement can be attributed to the practices of management staff in their organizations, who do not require them to provide periodic reports. As one participant put it:

I don’t think I’ve been asked to measure once or to provide one of my monthly reports that I do. I don’t think … I’m still yet to report that to anybody.

CP4

### Benchmarks Used in Social Media Measurement

Three types of benchmarks were apparent in social media measurement: personal benchmarking, comparative benchmarking, and metric benchmarking.

#### Personal Benchmarking

At least one participant alluded to using personal benchmarks to track progress, evaluate performance, and determine areas for improvement. This study conceptualized personal benchmarking as using self-set targets to evaluate social media performance. The adoption of personal benchmarks appears to be a convenient way to make up for the absence of an official one:

I gave myself KPIs because nobody gave me any.

CP2

#### Comparative Benchmarking

An alternative to personal benchmarking is comparative benchmarking. It involves measuring social media against those of best-in-class peers. One of the participants stated that:

My whole team attended Mayo Clinic’s Conference in Australia last year … in the next 5 years, we will like to be like Mayo clinic … We look at other organizations, for instance, the Royal Children’s Hospital has very good social media … We use other organizations as benchmarks and try to do better.

CP1

#### Metric Benchmarking

Unlike other types of benchmarking, metric benchmarking enables the numerical measurement of performance levels and comparison with set targets. According to an interviewee:

We’ve got quite huge targets that they [management] want us to achieve within the next 5 years of growing the page and reaching more people ... And they are very keen, and they monitor those results in their quarterly board meetings. So, they get a presentation every 3 months of where we are versus where we should be, and then they make recommendations based on that … So, we have very defined targets that we want to reach on a yearly basis, but then we work it, obviously, back to a monthly basis.

CP3

### Metrics Tracked in Social Media Measurement

Participants’ responses revealed three areas that they considered relevant indicators of their social media performance. These are reach, engagement, and conversion rates.

#### Reach

To determine the size of the audience that has encountered the social media posts targeted at them, health care organizations use the reach metric.

Social media reach is based on the number of followers, fans, subscribers, connections, and visibility [33], as illustrated by the following comments:

… we specifically measure audience size. So, [on] Facebook and Twitter and Instagram, how many followers do we have, and how fast is that growing?

CP3

I’ve got a metric that’s balanced towards being followed, which is good.

MD3

A reach metric allows health care organizations to estimate the proportion of an audience that sees a given social media message on a given social media platform.

If it’s [social media posts] reaching 2000 people, or 200,000 people saw it, we know even though they might not have clicked like, they still saw it, and they might have gotten some benefit out of it.

CP3

#### Engagement

By using engagement metrics, health care organizations can gauge the degree of audience interaction with their social media efforts, using public shares, likes, retweets, check-ins, and comments as indicators. According to one of the participants:

Every month we look at our average brand impressions and make sure we exceed that of the previous month. So, we set targets for ourselves. We also look out for metrics on our engagement and check whether we are meeting the targets. We always aim to surpass that of the previous month.

CP1

Another participant shared a similar view:

I have a look at how many people look at my blog, my articles on my blog, which is an automated stats collection.

MD5

There are several ways to measure how much interest a health care organization’s social media is generating, as noted below:

It could be the commentary, likes … it’s derived from an algorithm.

CP1

Another way to measure engagement is to use yardsticks, such as the Alexa rank.

According to an interviewee:

I’ll check my Alexa ranking every month or so just to make sure we’re going in the right direction. And if that has a massive turn, then I know that something isn’t right.

MD4

One of the alternatives to using yardsticks, such as the Alexa rank to measure engagement is to deploy an analytics tool to do the measurement. According to a participant:

…we have our Google Analytics running all the time on our website to be looking at traffic and so on. The regular posting on social media is designed to generate the traffic ... We track how successful they [social media] are at generating new and old traffic, how many clicks, how many people visit a certain number of pages after it, and so on. The bounce rate, things like that.

MD3

When health care organizations post relevant social media content, it encourages users to click through to the organization’s website. To specifically identify traffic from social media platforms, health care organizations deploy relevant metrics. One participant alluded to this by saying:

Every time we run a little campaign or a post, we put a sticky label on it to see what’s causing the traffic to rise or fall. So, we track which types of posts are most successful in driving more traffic through.

MD3

Another participant added that:

…we measure referrals back, so how many views on our website did we get from people who were using Facebook or Twitter or saw our stuff on Facebook or Twitter?

CP3

Measuring engagement also allows users to identify the platforms audiences are most interested in, the nature of their interaction with the platforms, and the geographical locations of the audience. In the words of a participant:

I measure the way in which people interact, including the platforms, the time zones, and the countries in which they interact. If there’s increase in the number of users in a certain country, then I can start thinking about translating contents into the language of that country.

MD4

#### Conversion

The conversion rate metric enables health care organizations to determine the percentage of visitors to their social media platform who donated to their social crowdfunding initiatives. In that context, a higher conversion rate is an indication of the success of social media initiatives. As one participant stated:

I think the amount of money we have raised through social media campaigns is an indication of success.

CP2

Health care organizations can also use the conversion rate metric to measure the percentage of people who attend an event after learning of the event on their social media platform. According to some interviewees, if many people attend their events after interacting with social media posts about those events, this demonstrates that their social media initiative has been successful. In the words of a participant:

[We consider our social media initiative successful] when patients come in and say we are here because we saw you on Facebook.

MD2

Another added:

If we have a function … we can do a paid advert for A$2,000 in a local paper and get 5 people in. If we do a A$500 advert on Facebook, we’ll get 50 people in, and the function is full, and we have to run additional events. So, from that, I suppose we can say we’ve been successful.

CP5

### Tools Used in Social Media Measurement

The study found that health care organizations used two types of tools for social media measurement: inbuilt analytics tools and add-on analytics tools.

#### Inbuilt Analytics Tools

Inbuilt analytics tools are embedded in the social media platform. For instance, Facebook page analytics is an inbuilt tool used to track user interaction on a Facebook fan page to improve understanding of the page’s performance. According to a participant:

Facebook Insights provides details on which posts have the most likes, comments, and shares, which means that page managers can see what content resonates with their audience and provide similar content to increase engagement with the page … Page Insights also provides basic demographic information about people who like your page, and this includes gender and age.

MD2

Another participant had the following to say about how they use inbuilt analytics tools for social media measurement:

... I will show them [management] monthly how many people were reached with the help of data obtained from Facebook Insights, and explain factors (humor, picture, videos, etc) that made the difference to audience engagement.

CP2

#### Add-on Analytics Tools

In contrast to inbuilt analytics tools, add-on analytics tools are not embedded in the social media platform. Rather, they are third-party software programs or scripts that are added to a social media platform to provide it with additional features and abilities. The additional capabilities of add-on analytics tools appear to have made them popular among health care organizations that use social media. According to an interviewee:

I’ll check my Alexa ranking every month or so just to make sure we’re going in the right direction.

MD4

In the words of another interviewee:

… we have our Google Analytics running all the time on our website, to be looking at traffic and so on.

MD3

A participant had this to say about the benefits of using Google Analytics:

I think that using Google Analytics is useful from a geolocation point of view. ... If there’s increase in number of users in a certain country, then I can start thinking about translating contents into the language of that country.

MD4

### Challenging Aspects of Social Media Measurement

Some of the feedback from participants indicated that there are aspects of social media use that health care organizations would like to measure but are currently unable to. Those identified specifically were health care social media’s conversion rate and its impact on both public health and patient satisfaction.

#### Conversion Rate

Although some of the interviewees alluded to measuring their social media conversion rate, it appears that many had more questions than answers. In the words of one particpant:

I wish there is a way to measure the follow-on. I can see how many people have gone to our website through Google Analytics, but the conversion rate is the problem. It’s hard to measure. I wish I could find out if we got more people as a result of our social media post.

CP2

Echoing a similar sentiment, another interviewee commented:

… we want the hard facts, we want to know who are we convincing [through social media] to come here rather than a competitor.

CP5

#### Impact on Public Health

Another aspect identified by some participants as difficult to measure is the impact of their social media activities on public health. In the words of one participant:

Like everything in health, what you will like to measure is whether you are making an impact in people’s health [through social media]… it’s hard to know.

MD2

The difficulty inherent in measuring the impact of social media interventions is particularly obvious at the aggregate level. One interviewee had this to say regarding the issue of measuring the global impact of social media-based health interventions:

There are many anecdotal pieces of information [regarding the impact of social media interventions] ... But how can you measure the globality of impact, because a lot of it is subconscious? It is difficult.

MD4

#### Patient Satisfaction

Finally, it is difficult to measure the extent to which patients are satisfied with the information health care organizations share with them on social media. In their words:

So you can have an exchange with them [patients], on social media, but how satisfied were they with service that we provided? … You can’t really measure the satisfaction that they got out of your exchange with them.

CP03

## Discussion

### Principal Findings

Five themes emerged from the analysis of health care organization participants’ responses regarding their approaches to social media measurement: frequency of social media measurement, the benchmark used for social media measurement, metrics tracked in social media measurement, tools used in social media measurement, and challenging aspects of social media measurement. Table 1 presents a summary of the findings. The analysis presented in this section elucidates responses to the research question—how do health care organizations approach social media measurement?

**Table 1:** Principal findings: themes and categories.

Themes	Categories
Frequency of measurement	Frequently Infrequently Never
Benchmarks used in measurement	Personal benchmarking Comparative benchmarking Metric benchmarking
Metrics tracked in measurement	Reach Engagement Conversion
Tools used in measurement	Inbuilt analytics tools Add-on analytics tools
Challenging aspects of measurement	Conversion rate Impact on health Patient satisfaction

### Frequency of Measurement

The study observed a discrepancy among participants in the frequency of social media measurement. While some health care organizations measured their social media frequently, others measured infrequently or not at all.

It appears that the frequency of social media measurement depended on the accountability expectations of management. Health care organizations whose management demand a formal report of their social media performance tend to measure their social media frequently. Whereas health care organizations whose management does not demand accountability from those who run their social media tend not to measure their social media at all, or they measure them infrequently.

Accountability expectations were influenced by whether the management trusted that their social media was performing a useful health service. Not measuring social media or infrequently measuring them could be an indication that managers of a health care organization are not fully aware of the usefulness of using social media in health care settings.

Further, it appears that medical doctors were less likely to measure social media regularly compared with communications personnel, perhaps due to their heavy workloads or limited experience using social media for business.

Social media should be tracked frequently, that is, weekly or monthly [34]. Health care organizations that frequently measure their social media can track how their social media initiatives are progressing vis-à-vis their target for the month, which allows them to address any issues that could impede their ability to meet set targets. Measurement needs to be an ongoing cycle [35]. Measuring social media on an ongoing basis would enable an understanding of the extent to which social media has supported the realization of set objectives [19,36-38].

### Benchmarks Used in Measurement

Although many health care organizations use benchmarks to measure their social media performance, others do not. Benchmarks are standards against which performance is compared; thus, they enable social media users to know which areas require more attention [34]. The types of benchmarking that were apparent from the study are personal benchmarking, comparative benchmarking, and metric benchmarking.

Some health care organizations that use social media do not have an official benchmark against which they measure their performance; hence, some users have set personal benchmarks in the absence of an official one. Personal benchmarks are standards individuals set for themselves that allow them to track their progress and evaluate themselves to determine how they need to improve. The standards are set regarding what is important to individuals, thereby guiding them against irrational actions [39]. Setting a personal benchmark could help motivate health care organizations to perform better because it enables them to measure their performance.

An alternative approach used by some of the more ambitious health care organizations to measure their social media is comparative benchmarking. Health care organizations that used comparative benchmarking tracked their social media operations and compared their results with those of more established health care organizations. In this context, it can also be referred to as peer benchmarking. In health care settings, peer benchmarking is used when there is a need to raise performance levels to be on par with the performance of leaders in the field [40]. In doing so, individual results may be used to compare with peer results [41]. Comparative benchmarking has the potential to support health care organizations that are neophytes in their efforts to improve their social media performance. It encourages them to strive to be like more established health care social media users in the industry or region.

Health care organizations that have clear social media goals may prefer metric benchmarking to both personal benchmarking and comparative benchmarking. Metric benchmarking involves the use of statistical procedures to evaluate performance against set targets [42]. Consequently, social media use is data-driven, with quantitative performance improvement objectives that are predictable and align with the needs of the health care organization, thereby ensuring a more objective appraisal of social media performance.

### Metrics Tracked in Measurement

It was noted that metrics tracked by health care organizations that use social media include reach, engagement, and conversion rates.

Many health care organizations use reach metric to measure their social media performance because it is easy to calculate. Social media reach is an estimate of the number of users that could have contact with a social media post [33]. It is the aggregate of the audience of a social media platform, including subscribers and visitors [33]. The reach metric is informative, given that it allows a health care organization to obtain a better understanding of their audience and the geographical regions that their posts or content reach. An expanding audience would indicate to a health care organization that their online presence is growing.

A wide reach does not necessarily translate to deep engagement. In other words, it is possible for a health care organization’s reach via social media to be wide yet only able to engage a small proportion of its target audience. Consequently, there is a need for the engagement metric. The engagement metric measures the ability of a social media user to establish dialogue and interaction with other users [37,43]. Measuring engagement allows health care organizations to know who is reading their social media posts, the content that interests users, and the platform that is popular with users. Social media data, such as the number of “likes,” “fans,” or “shares” for Facebook, or the number of “tweets,” “retweets,” or “replies” for Twitter are used to compute engagement [43]. The type of indicator required depends on the specific social media platform. For instance, engagement can be calculated by counting the number of replies on Twitter, the number of comments on Facebook, and the number of subscribers on YouTube [37,43]. More shares, likes, or comments for a health care organization’s social media posts would indicate that their message resonates with their audience.

Although a high level of engagement indicates that the audience finds social media efforts interesting, it is not a confirmation that they are taking the desired action. To be sure that their social media posts are influencing the behavior of their audience, health care organizations use the conversion metric. Social media conversion rate is a measure of the percentage of the audience who take the desired action after interacting with social media content [44]. Health care organizations use conversion metrics to measure the number of people that respond to their call-to-action on social media. When a health care organization is seeking to motivate action on the part of its audience, tracking the conversion rate allows them to monitor the percentage of users who take the recommended action. Participants reported that the conversion metrics helped them to know the proportion of people who visited their social media pages that donated to their crowdfunding initiatives. Ultimately, the indicators used to measure conversion rates vary depending on the recommended action. For instance, if the recommended action is for the audience to donate to a crowdfunding initiative, the indicator would be the number of people that donated after finding the campaign on social media. Similarly, if the recommended action is that the audience attends an event organized by the health care organization, the indicator would be the number of people that arrive at the event after viewing the invitation on social media.

### Tools Used in Measurement

It was noted that when it comes to measuring health care social media, both inbuilt analytics tools and add-on analytics tools are useful.

Inbuilt analytics tools are measurement tools built into most social media platforms [45]. For example, Facebook Analytics provides a general overview of a user’s Facebook page, their audience, and the performance of their posts [45]. Data taken from one day, the previous week, or the last month can be drilled-down to reveal more high-level statistics [45]. It shows an organization the performance of their posts and the behavior of their followers, thus allowing them to identify the best time of day to post, the best day of the week to post, and the most popular type of content to post [45]. Other examples of inbuilt analytics tools include Pinterest Analytics, Twitter Analytics, Instagram Analytics, and YouTube Analytics [46].

Inbuilt analytics tools are popular because there are no acquisition costs [45,47]. Hence, they are suitable for health care organizations that have a relatively small social media budget and health care organizations that do not use social media extensively or use only one social media platform. The main limitations of inbuilt social media analytics tools are that they are usually only able to support individuals or small brands [47] and specific social media accounts [45].

Health care organizations that are mature social media users and own multiple social media platforms may prefer to use add-on social media analytics tools to appraise their social media. Add-on social media analytics tools include computer software services such as Google Analytics and Alexa ranking that can be added to social media to enable the tracking of relevant metrics. Users can employ the Google Analytics tool to sift and sort visitors to social media platforms with dimensions such as location. As an analytics tool, it can globally track social media and other online activities [45]. Google Analytics could work well with social media platforms. To optimize usage, the user must install both Google Analytics and Google Tag Manager before tagging the aspect of social media they would like to measure [48]. Google Tag Manager enables data and metrics from relevant social media websites to be sent to Google Analytics for analysis [48]. Alexa rank, on the other hand, is a measure of the popularity of a website or a social media site in terms of traffic [49]. It is calculated using a proprietary methodology that combines a site’s estimated traffic and visitor engagement over some time [50].

Other examples of add-on analytics tools include BuzzSumo, Vizia, SumAll, and Quintly [46]. Add-ons are arguably easier to use than inbuilt analytics tools. They are cross-platform and are particularly relevant for managing multiple social media platforms and accounts [45-47]. Users can use them to track all their social media platforms simultaneously, thereby enhancing efficiency and ensuring more consistent and valuable results [45]. The additional features mean that most of these tools entail additional costs. That notwithstanding, they are the most suitable analytics tools for health care organizations that use several social media platforms.

### Challenging Aspects of Measurement

Information technology creates value but identifying where, how, and how much value can be problematic [51]. Social media has considerable potential to make a positive contribution to health care; however, results from its use are abstruse [52]. One of the most critical issues in the appraisal of information systems investments is the question of what to measure [53]. Although many participants alluded to measuring their social media, many did not appear confident that they were successful with certain aspects of measurement.

Participants identified conversion rate, impact on public health, and patient satisfaction as areas that were difficult to measure. Although some health care organizations found the conversion metrics useful for tracking audience responses to social crowdfunding initiatives, they appeared to have unanswered questions regarding using the metric in different contexts.

It was also noted that health care organizations would like to know the impact their social media initiatives are having on the broader society in terms of disease prevention, prolonging life, and promoting human health. However, according to the results of the study, participants do not know how to ascertain their impact. That is not surprising because the societal impact of information technology is difficult to conceptualize, and any conceptualization is likely to be subjective [54].

Participants also alluded to being interested in learning the level of patient satisfaction with their social media sites, but being unable to measure it. Patient satisfaction is an important and commonly used indicator for measuring the extent to which patients are content with the health care they have received from their health care organization [55]. Given that patient satisfaction affects clinical outcomes, patient retention, and medical malpractice claims [55], it is logical that health care organizations that use social media would be keen to know the extent to which patients are happy with their social media sites.

The ongoing social media measurement challenges highlight the need for a transparent, standardized, and flexible measurement framework [19]. The lack of a consensus approach concerning certain aspects of measurement suggests that social media measurement in health care settings is at a nascent stage. It seems that the health industry lags behind other industries in terms of knowledge, sophistication, and integration of social media strategy.

### Strengths and Limitations

This study sheds light on the social media capabilities of health care organizations. It demonstrates the yardsticks that are effective for social media measurement as well as potential blind spots for which suitable yardsticks appear to be elusive. By exploring the merits and limitations of current techniques used to appraise social media in health care settings, this study provides information with which to revise and improve the existing measurement criteria.

A health care organization’s ability to measure their social media, among other things, reflects their level of social media maturity [18]. By investigating how health care organizations in Australia approach social media measurement, this study reveals participants’ level of social media maturity. Thus, the results of this study can help health care organizations take stock of their social media capabilities and determine which strategies are appropriate for their maturity level and for optimizing success [18].

Moreover, measurement is a critical success factor of social media initiatives [56]. Thus, by identifying the approach used by health care organizations in social media measurement, this study enables a deeper understanding of some of the tools and techniques required for successful social media campaigns.

Despite the contributions of this study to the growing body of research on the use of social software in health care settings, several important limitations need to be considered. First, the results of this study should be interpreted as indicative and not necessarily generalizable, considering that the study was restricted to Australia. Second, given that only medical doctors and the communications personnel of health care organizations were interviewed for this study, a research sample with more diverse profiles may suggest additional themes. Last, it is important to note the time frame of this study when considering its findings, since usage and attitude toward social media evolve rapidly.

### Conclusions

This qualitative study provides insight into how health care organizations approach social media measurement. Although many participants showed great interest in using various tools and techniques to measure their social media, they still had some unanswered questions. Despite the availability of tools that enable users to track social followers and click-through, measuring the effectiveness of social media initiatives remains a challenge [21]. While many online activities can be appraised using defined quantitative metrics, social media, among other things, generates qualitative data, which traditional metrics alone cannot effectively measure [19]. This measurement problem is exacerbated by the lack of an overarching measurement approach, which causes difficulties for organizations wanting to prove the usefulness of their social media [19,57,58].

Without the ability to define and measure the use of social media, it will be difficult to derive value from them [21,59]. Therefore, the challenge for health care organizations that use social media is to determine what to measure and the data requirement of such measurement. A comprehensive and consistent measurement approach for social media would help improve its use.

Health care organizations should work towards improvements in how to use social media, and how to measure their effectiveness. This study calls for social media training in health care organizations. Such training must focus on both how to use relevant tools and how to effectively measure their use.

## Appendix

Multimedia Appendix 1 Interview Guide.

## Abbreviations

CP: communications personnel
MD: medical doctor
