# Supplementary material for: How Health Care Organizations Approach Social Media Measurement: Qualitative Study
Source: JMIR Form Res. 2020 Aug 14;4(8):e18518. doi: 10.2196/18518 (PMC7455874; doi:10.2196/18518)
Supplement: Multimedia Appendix 1 [file formative_v4i8e18518_app1.pdf]

Interview Guide

PROJECT: SOCIAL MEDIA MEASUREMENT IN HEALTHCARE SETTINGS

Interview N°: N°<br>Date:<br>Place:

**OPENING REMARKS**
• Purpose of the interview<br>• Time frame<br>• There are no right or wrong answers<br>• Questions?

**QUESTIONS**
How do you define success in social media?
Please describe your organization's social media strategy
How do you ascertain that your social media strategy is hitting the mark?
How often do you measure your social media?
What yardstick do you use to measure the effectiveness of your social media efforts?
How do you track your social media performance?
What tools do you use for social media measurement?
In your opinion, what are the challenging aspects of social media measurement?
